# The baseline distribution of malaria in the initial phase of elimination in Sabang Municipality, Aceh Province, Indonesia

**DOI:** 10.1186/1475-2875-11-291

**Published:** 2012-08-21

**Authors:** Puji BS Asih, Ismail E Rozi, Herdiana, Nandha R Pratama, Anggi PN Hidayati, Sylvia S Marantina, Sully Kosasih, Krisin Chand, Suradi Wangsamuda, Faisal A Rusdjy, Maria E Sumiwi, Ali Imran, Titik Yuniarti, Tahi Sianturi, Jamilah Nur, Asnita, Bukhari, Cut Barussanah, Muhammad Yani, Cut Ainun, Kurnia Jamil, Cut Mariam, Simon P Sengkerij, Ferdinand J Laihad, William Hawley, Din Syafruddin

**Affiliations:** 1 Eijkman Institute for Molecular Biology, Jalan Diponegoro, 69, Jakarta, 10430, Indonesia; 2 UNICEF, Jakarta, 12920, Indonesia; 3 Office Health Department, Sabang Municipality, Aceh Province, Indonesia; 4 Provincial Health Laboratory, Aceh Province, Indonesia; 5 Department of Internal Medicine, Faculty of Medicine, University of Syah Kuala, Syah Kuala, Indonesia; 6 Department of Parasitology, Faculty of Medicine, University of Syah Kuala, Syah Kuala, Indonesia; 7 Department of Parasitology, Faculty of Medicine, Hasanuddin University, Makassar, 90245, Indonesia

## Abstract

**Background:**

Sabang Municipality, in Aceh Province, Indonesia, plans to initiate a malaria elimination programme in 2013. A baseline survey of the distribution of malaria in the municipality was conducted to lay the foundations for an evidence-based programme and to assess the island’s readiness to begin the elimination process.

**Methods:**

The entire population of the municipality was screened for malaria infection and G6PD deficiency. Specimens collected included blood slides, blots and tubes for selected households.

**Results and Discussion:**

Samples were collected from 16,229 residents. Microscopic examination of the blood smears revealed 12 malaria infections; 10 with *Plasmodium falciparum* and 2 with *Plasmodium vivax*. To confirm the parasite prevalence, polymerase chain reaction (PCR) diagnosis was performed on the entire positive cases by microscopy and randomized 10% of the microscopically negative blood samples. PCR revealed an additional 11 subjects with malaria; one *P. falciparum* infection from the village of Paya Keunekai, and nine *P. vivax* infections and one mixed *P. falciparum*/*P. vivax* infection from the village of Batee Shok. The overall slide positivity rate was 0.074% (CI 95%: 0.070 – 0.078) and PCR corrected prevalence 0,590% (CI 95%: 0.582 – 0.597). Analysis of 937 blood samples for G6PD deficiency revealed two subjects (0.2%) of deficient G6PD. Analysis of several genes of the parasite, such as *Pfdhfr*, *Pfdhps*, *Pfmdr1*, *Pfcrt*, *Pfmsp1*, *Pfmsp2*, *Pvdhfr*, *Pvdhps*, *Pvmdr1* and host gene, such as G6PD gene revealed that both *P. falciparum* and *P. vivax* carried the mutation associated with chloroquine resistance.

**Conclusion:**

Malariometric and host genetic analysis indicated that there is a low prevalence of both malaria and G6PD deficiency in the population of Sabang Municipality. Nevertheless, malaria cases were clustered in three rural villages and efforts for malaria elimination in Sabang should be particularly focused on those three villages.

## Background

Malaria remains a major public health problem in Indonesia, with 30 million cases and 120,000 deaths annually. Recently measures of annual parasite incidence (API) have varied substantially between provinces, but the highest API is consistently detected in the eastern parts of Indonesia. Aceh Province, Sumatera, is the western most point of the archipelago, and malaria has been documented throughout. However, there have been few published reports of malaria prevalence in Sumatera in general and in Aceh in particular. Malaria is endemic in 21 districts and municipalities in Aceh Province, where the annual malaria incidences (AMI) in 2003 and 2004 were 4.94% and 3.2% respectively [1]. Malaria prevalence surveys in 2005 and 2006 in 11,763 subjects from 3,771 households in five districts along the tsunami-affected western coastline reported a slide positivity rate for all *Plasmodium* species of 2.1% [2].

Malaria in Sabang has been documented by several previous surveys both before and after the 2004 tsunami that destroyed much of the coastal region in Aceh [1]. Resistance to chloroquine and sulphadoxine-pyrimethamine, two historical anti-malarial mainstays, was reported in an *in vitro* study conducted during 1984–1985 [3], findings which were supported by an *in vivo* study in 2004 [4]. The AMI of Sabang Municipality fell from 269 cases per 1,000 populations in 2001 to 33 cases per 1,000 populations in 2009, while the API similarly fell from 101 cases in 2001 to 3 cases per 1,000 populations in 2009 [3]. Malariometric surveys in the villages of Suka Jaya and Paya Seunara found that the slide positivity rates decreased from 7.4% in 2005 to 5.4% in 2006. Measurement of the prevalence of glucose-6-phosphate dehydrogenase (G6PD) deficiency using a colorimetric assay identified 0.8% subjects with less G6PD activity, and molecular analyses found Vianchang type G6PD deficiency mutation (Syafruddin, unpublished report).

The Indonesian Ministry of Health announced a country wide malaria elimination policy in 2009 that envisioned a combat against the disease lasting from 2010 to 2030. Aceh Province aims to eliminate malaria by 2015 but for Sabang Municipality, which includes the islands of Weh, Rondo and Rubiah, the target for malaria elimination is 2013 [5]. An optimal strategy for the elimination programme will be based on a contemporary reassessment of the malaria situation as well as the G6PD prevalence in the islands. Appropriate knowledge on G6PD prevalence is important, haemolysis may be induced during primaquine treatment among G6PD-deficient individuals. To accomplish this, a study was conducted in Sabang Municipality to obtain baseline data on malaria prevalence as well as host genetic factors, such as G6PD deficiency, that may contribute to malaria morbidity.

## Methods

### Study site

**Figure 1:**
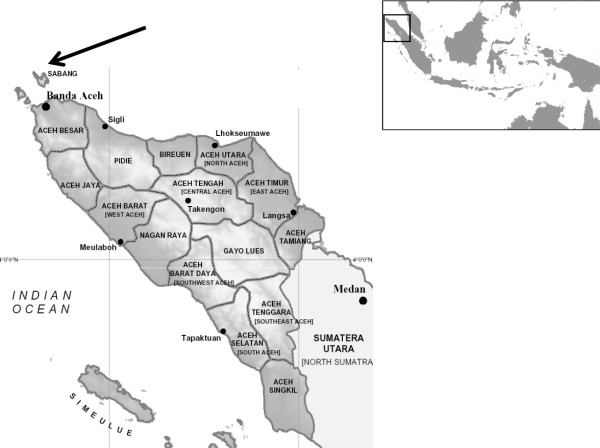
**Aceh Province with Sabang Municipality indicated by the arrow and location within the Indonesian archipelago (inset).** Not to scale.

Sabang Municipality is administratively included in Aceh Province, Indonesia. It is located at latitude 5° 49' 30" north and longitude 95° 18' 28" east (Figure 1). It occupies an area of 4,051 sq km, and in 2009 its total population was 24,815. The rainy season usually occurs from December to April, during which time the temperature centres around 18–20°C. Dry season temperatures are around 25-33°C.

### Malariometric and G6PD surveys

**Figure 2:**
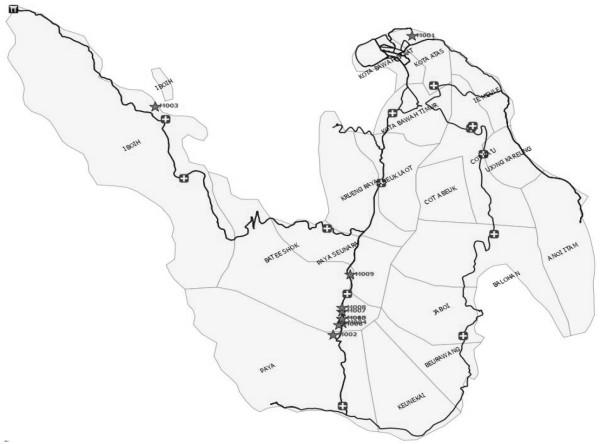
**Malaria case (★) distribution and Primary Health Center (+) in Sabang Municipality.** Not to scale.

Malariometric and G6PD surveys were conducted from May to October 2010, in 14 villages with known malaria transmission. Urban areas with no recorded transmission were excluded from the survey. Malariometric surveys include interview, physical examination of malaria symptoms and signs, body weight and axillary temperature. For each individual, thick and thin blood smears were collected along with a blood spot for PCR on filter paper and whole blood drops for G6PD test and haemoglobin (Hb) tests. All slides were reviewed by an expert microscopist. Slide positive cases were confirmed with PCR. In addition, 10% of negative cases were screened with PCR, and all blood samples from villages with microscopically positive cases. Selection of the 10% negative samples was done through computer programme. All negative samples in each village were listed sequentially in microsoft excel. A Random option was used to generate sample sequence and the samples were selected in ten-fold fashion. At the end, 1,725 samples were proportionally selected. Locations of confirmed positive cases were geocoded with GPS (Figure 2). For G6PD test, blood were directly dropped onto a 1.5 ml microtube containing 760 μl of water, 20ul of substrate mixture and 20 μl of dye mixture of G6PD assay kit. The tube was then vigorously shaked for 5 sec and incubated at 25-37°C for 20–30 minutes. The reaction was stopped by adding 10 μl of 1 mol/l HCL and compared the developed color with those of positive and negative controls. This method used water-soluble tetrazolium salt, WST-8, that also produces water-soluble formazan (Dojindo Laboratories, Tokyo, Japan). The G6PD enzyme activity was divided into five categories: A: 0% activity (G6PD-deficient), B: 25% activity, C: 50% activity, D: 75% activity and E: 100% activity (normal activity). The sample selection for G6PD screening was based on households in urban village of Cot Bau where the population of the entire municipality was well represented. In each selected household, chief of the household and his wife were enrolled. The total subjects enrolled in G6PD survey was 937 subjects and represented over 50% of the total households in this village. To determine the Hb status, a portable digital haemocytometer (Haemocue AB Hb201+, Angelholm, Sweden) was used.

### Laboratory methods

Thick and thin blood films were stained with Giemsa and examined using 1,000X oil immersion light microscopy. At least 200 ocular fields were examined before considering a slide negative, and parasite densities were counted as parasites per 200 leukocytes and reported as parasites/mm^3^ assuming a white blood cell count of 8,000/mm^3^[6].

### Extraction of DNA

Parasite and human host DNA was extracted from blood samples using chelex-100 ion exchange (Biorad Laboratories, Hercules, CA, USA) according to a procedure described previously [7]. The DNA was either used immediately for PCR amplification or stored at −20°C for later analysis.

### Molecular analyses on the parasite and host

Molecular analyses were performed using PCR amplification, restriction fragment length polymorphisms (RFLP), and sequencing on several genes of the parasite such as *Pfdhfr*, *Pfdhps*, *pfmdr1*, *pfcrt*[8-10], *Pfmsp1*, *Pfmsp2*[11,12], *Pvdhfr*, *Pvdhps*[13,14], *Pvmdr1*[15] and host gene such as G6PD gene [16]. The PCR reactions were carried out in a condition similar to the previously published report [8-16]. PCR diagnosis to further confirm the parasite rate on the entire positive cases was performed using 18S rRNA gene primer in a condition similar to the previously published report [17].

## Results

### Malariometric surveys

In all, 16,229 individuals were surveyed from 14 villages. The surveys covered 83.47% of the total inhabitants of the targeted survey areas and by microscopy 10 subjects were found with *Plasmodium falciparum* infection: seven from the village of Batee Shok, two from Paya Keunekai and one from Ie Meuleu. *Plasmodium vivax* malaria was found in one subject from Iboih and one from Rondo island. PCR testing was performed on seven malaria falciparum slide positive cases and one malaria vivax slide positive case (blood blots were unavailable for three malaria falciparum slide positive cases and one malaria vivax slide positive case). PCR (10% of negative cases and all blood samples from villages with microscopically positive) revealed an additional 11 malaria-positive subjects: one mixed *P. falciparum*/*P. vivax* infection from Batee Shok, one *P. falciparum* from Paya Keunekai village, and nine *P. vivax* from Batee Shok village (Table 1 and 2). The slide positive rate was 0.074% (CI 95%: 0.070 – 0.078) and PCR corrected 0.590% (CI 95%: 0.582 – 0.597). Of those that were positive for malaria by microscopy, 9 (75%) had clinical symptoms such as fever and/or history of headache. The remaining 14 subjects, all from the village of Batee Shok, had neither symptoms nor history of recent fever. Overall, of the total 23 positive cases by microscopy and PCR, 17 (73.9%) cases were found at Batee Shok and the remainder were found in Iboi, Paya Keunekai, Ie Meulee and Rondo island. However, malaria prevalence peaked in the village of Batee Shok, which harboured 17 malaria-positive subjects among a total 1,109 subjects examined (1.53%).

**Table 1:** Malaria positives by village in Sabang Municipality

	**Detection method**					
**Location in Sabang**	**Microscopy**			PCR*		**Total**
	Pf	Pv		Pf	Pv	
Iboih	-	1		-	-	1
Batee Shok	7	-		1	9	17
Paya Keunekai	2	-		1	-	3
Ie Meulee	1	-		-	-	1
Rondo Island	-	1		-	-	1
**Total**	10	2		2	9	23

* = 10% of negative cases and all blood samples from villages with microscopically positive.

**Table 2:** Number of PCR positives samples from different groups

**No**	**Sample sources**	**Total PCR**	**PCR Results**		
			**Pf**	**Pv**	**Mixed**
1	Microscopy positive subjects	8*	10	2	0
2	Villages with microscopy positive				
	Iboih	284	0	0	0
	Batee Shok	898	0	9	1
	Paya Keunekai	308	0	1	0
3	10% random sample microscopy negative from cencus data in each village	1,725	0	0	0
	Total	3,223	10	12	1

Total PCR corrected prevalence = 0.590% (19/3223) (CI 95%: 0.582 – 0.597).

Total microscopy prevalence = 0.074% (12/16229) (CI 95%: 0.070 – 0.078).

(*) 4 of 12 samples have no blood blots.

### Prevalence of G6PD deficiency

The head of each household and his wife were enrolled in the G6PD survey. Of the total 937 subjects enrolled, two (0.2%) tested positive for G6PD deficiency. Analysis for the presence of the most commonly found mutations: Mediterranean (563C/T), Coimbra (592 C/T), Vianchang Canton (1376 G/T), Vanua Lava (383 T/C), Mahidol (487 G/T), Chatam (1003 G/A), Kaiping (1388 G/A) and Vianchang (871 G/A, 1311 C/T) found none of these in the two positive subjects.

### Haemoglobin status

A total of 16,229 subjects was analysed for weight and Hb levels. The mean of Hb level was relatively high in each age group except for the below five years, which had a Hb level slightly above or below the borderline (anaemia cut-off for Hb level is 11 g/dL). Distribution of age, children, sex and mean of Hb level per village also indicated relatively high in each group. However, due to the low prevalence of malaria, the relationship between anaemia and malaria could not be determined. Malnutrition was found in one village Kreung Raya (Additional files 1, 2, 3, 4, and 5).

### Genotypic profiles of *Plasmodium falciparum* isolates

Molecular analysis of the parasites collected throughout the survey found the 76 T allele of the *pfcrt* gene, a molecular marker for parasite resistance to chloroquine (Table 3). The proportion of the isolates carrying the 86Y polymorphism of the *pfmdr1* gene was 37.5%. The 1042D polymorphism of *pfmdr1* was detected in one isolate. No polymorphisms at codons 1032 and 1246 of the *pfmdr*1 gene were observed in any of the isolates examined. Amplification of the *dhfr* gene indicated that the majority of the isolates carried the mutant 108 N allele. 108T, 59R, 16V, 50R, 51I, 164L, 436A 437G, 540E, 581G and 613 S/T polymorphisms were not detected in any of the isolates examined. Analysis of the parasite genotype indicated infection with multiple parasite strains (Table 3). All samples carried the 3D7 type of which three samples were mixed with the FC27 type. Analysis on *MSP1* gene revealed the K1, MAD20 and RO33 types but three samples could not be determined.

**Table 3:** **Genotypic profile of *Plasmodium falciparum***

**Gene analysed**													
**Sample Code**	***PfMSP2***		***PfMSP1***			***PfCRT***	***PfMDR1***		***PfDHFR***			***PfDHPS***	
	**3D7**	**FC27**	**KI**	**MAD20**	**RO33**	**K76T**	**N86Y**	**N1042D**	**A16V**	**C59R**	**S108N/T**	**A437G**	**K540E**
ASK10057	+	--	+	+	--	T	Y	N	A	C	N	A	K
ASK20503	+	+	--	--	--	T	N	N	A	C	S/N	A	K
ASK20505	+	--	+	--	--	T	N	N	A	C	N	A	K
ASK20512	+	--	--	--	--	T	Y	N	A	C	N	A	K
ASK20535	+	--	--	--	--	T	N	N	A	C	S	A	K
ASJ10083	+	+	--	+	--	T	Y	N	A	C	N	A	K
ASJA0324	+	--	--	+	--	T	N	N	A	C	N	A	K
MC-HN	+	+	+	--	+	T	N	N/D	A	C	S/N	A	K

No blood blots for three samples.

### Genotypic profiles of *Plasmodium vivax* isolates

Molecular analysis of the parasites collected throughout the survey indicated that the 976 F allele of the *pvmdr1* gene, a molecular marker for resistance to chloroquine, was found in 10 of 11 isolates examined. Amplification of the *Pvdhfr* gene indicated that 63.6% of the isolates carried the wild type 117S allele and the reminder carried the 117 T. Mixed allelic infection of 117 T/N was found in one isolate. Analysis of codon 57 and 58 of the *Pvdhfr* gene revealed three isolates carried the 57 F allele and five isolates carried the 58S allele, three of which are mixed allelic infection with 58R. No polymorphisms were observed at codons 13 or 16 in any of the isolates examined. Furthermore, no polymorphisms were observed at codons 383 and 553 of *Pvdhps* gene in any of the isolates examined in this study (Table 4).

**Table 4:** **Genotypic profile of *Plasmodium vivax***

**No Sample**	***Pvmdr1***	***PvDHFR***					***PvDHPS***	
	**Y976F**	**S117T/N**	**F57I/L**	**I13L**	**T61M**	**S58R**	**A383G**	**A553G**
ASK20097	F	S	L	I	M	S	G	A
ASK20143	F	S	L	I	M	R	G	A
ASK20260	F	T	L	I	M	R	G	A
ASK20328	F	S	L	I	M	R	G	A
ASK20334	F	S	L	I	M	R	G	A
ASK20483	F	T	L	I	M	S	G	A
ASK20712	F	S	L	I	M	S + R	G	A
ASK20747	F	S	F	I	M	R	G	A
ASK20803	F	T + N	F	I	M	S + R	G	A
ASK20953	F	T	L	I	M	S + R	G	A
ASJ10148	Y	S	F	I	M	R	G	A

No blood blots for one sample.

## Discussion

Mass blood surveys conducted in three periods between May and October 2010 describe a low prevalence of malaria in Sabang Municipality. *P. falciparum* and *P. vivax* were found, with *P. falciparum* predominant. The findings that most of the malaria-positive subjects had clinical symptoms suggest that malaria endemicity in the island is relatively unstable. Nevertheless, the relatively high proportion of asymptomatic, sub-patent (microscopy negative and PCR positive) infection alerts to the need of a more reliable detection method as the cases might be potential source of infection as that found in South America [18]. Malaria cases were found in only three rural villages, corroborating previous observations [4]. It is highly recommended that efforts be concentrated on containing malaria in this village through active and passive case-finding and prompt, appropriate treatment.

Genotyping found that all of the *P. falciparum* isolates examined carried chloroquine-resistant alleles. All of the isolates examined carried the 76 T mutant allele of the *pfcrt* gene and in addition 30% of isolates carried the 86Y allele of the *pfmdr1* gene. This result is in line with previous observations that the parasite on this island is resistant to chloroquine [1]. In addition, among the *P. vivax* isolates examined, 10 carried the 976 F allele of the *pvmdr1* gene that has been associated with chloroquine resistance [9,11]. Both *P. falciparum* and *P. vivax* in this island are resistant to chloroquine, providing a rational basis for the use of arteminisin-based combination therapy for any malaria cases found.

Analysis of the *Pfdhfr* gene also indicated a high proportion of the *P. falciparum* isolates carry mutant alleles associated with resistance to pyrimethamine. However, the frequency with which the mutant allele of *Pfdhfr* gene was found is slightly lower than that of a previous study done in 2006, likely due to the larger sample size of our study. *Pfdhps* alleles associated with sulphadoxine resistance were not found in any of the isolates examined, indicating that the parasites on this island are still sensitive to treatment with sulphadoxine-pyrimethamine, quintuple mutations in *dhfr* and *dhps* being associated with sulphadoxine-pyrimethamine treatment failure [12].

The prevalence of G6PD-deficiency cases was also low (0.2%) among the 937 subjects screened. These subjects represent households and the prevalence reflects a larger population, but the G6PD deficiency prevalence found in this survey is much lower than that found in a previous survey (0.8%) that was conducted 2006 (unpublished observation). The difference might be attributed to the much higher number of subjects involved in this study. In the previous G6PD survey, the Vianchang mutation (871 G/A, 1311 C/T) was found in one subject but during the current survey none of the most common G6PD mutations were found among the G6PD-deficient cases.

## Conclusions

This combined malariometric and host genetic analysis indicated that both malaria and G6PD deficiency exist in low prevalence among the examined population in Sabang Municipality. In the context of the malaria elimination programme, several rational actions are implied; i) more flexible use of primaquine, the only available anti-malarial for antigametocyte and radical cures; ii) to contain malaria transmission in this island, a limited mass drug administration could be performed at the three villages where the malaria cases was detected; and iii) active case monitoring and treatment on migrants should be prepared.

## Competing interests

The authors declare that they have no competing interests.

## Authors’ contributions

PBSA, IEPR, NRP, APNH, SSM, SK and DS performed samples collection, molecular assays, data analysis, and manuscript writing. KC, SW, FAR, MES, AI, TY, TS, JN, A, B, CB, MY, CA, KJ, and CM collected field samples. AI, H, MES, SPS, FL, WH, and DS designed the study was responsible for management and fund raising for this study. All authors read and approved the final manuscript.

## Financial support

This study was funded by UNICEF Indonesia.

## Supplementary Material

Additional file 1 Detailed number of MBS subjects and coverage percentage of enrollment. Click here for file

Additional file 2 Distribution of respondents by age group. Click here for file

Additional file 3 Descriptive Statistic for Hb level by village. Click here for file

Additional file 4 Distribution of anaemic status by village. Click here for file

Additional file 5 Age distribution of participants in MBS divided per villages. Click here for file
