# Supplementary material for: The baseline distribution of malaria in the initial phase of elimination in Sabang Municipality, Aceh Province, Indonesia
Source: Malar J. 2012 Aug 21;11:291. doi: 10.1186/1475-2875-11-291 (PMC3478225; doi:10.1186/1475-2875-11-291)
Supplement: Additional file 1 — Detailed number of MBS subjects and coverage percentage of enrollment. [file 1475-2875-11-291-S1.doc]

Additional file 1. Detailed number of MBS subjects and coverage percentage of enrollment

| No | Village | Census Data |  | Total Enrolled Subjects | |  | Total Un-examined Subjects | |
| --- | --- | --- | --- | --- | --- | --- | --- | --- |
|  |  | n |  | n | % |  | n | % |
|  |  |  |  |  |  |  |  |  |
| 1 | Iboih | 711 |  | 546 | 76,8 |  | 165 | 23,2 |
| 2 | Batee Shok | 1.109 |  | 948 | 85,5 |  | 161 | 14,5 |
| 3 | Paya Seunara | 1.812 |  | 1.189 | 65,6 |  | 623 | 34,4 |
| 4 | Kreung Raya | 989 |  | 517 | 52,3 |  | 472 | 47,7 |
| 5 | Aneuk Laot | 980 |  | 739 | 75,4 |  | 241 | 24,6 |
| 6 | Kota Bawah Timur | 1.999 |  | 1.572 | 78,6 |  | 427 | 21,4 |
| 7 | Paya Keuneukai | 394 |  | 312 | 79,2 |  | 82 | 20,8 |
| 8 | Keuneukai | 709 |  | 638 | 90,0 |  | 71 | 10,0 |
| 9 | Jaboi | 685 |  | 502 | 73,3 |  | 183 | 26,7 |
| 10 | Balohan | 2.295 |  | 1.682 | 73,3 |  | 613 | 26,7 |
| 11 | Cot Abeuk | 641 |  | 459 | 71,6 |  | 182 | 28,4 |
| 12 | Cot Ba'u | 3.670 |  | 3.138 | 85,5 |  | 532 | 14,5 |
| 13 | Anoi Itam | 639 |  | 410 | 64,2 |  | 229 | 35,8 |
| 14 | Ie Meulee | 2.810 |  | 1.810 | 64,4 |  | 1.000 | 35,6 |
| 15 | Rondo island | - |  | 1 | - |  | - | - |
| 16 | Schools and Offices | - |  | 1.766 | - |  | - | - |
| Total | | 19.443 |  | 16.229 | 74,0 |  | 4.981 | 26,0 |
|  | |  |  |  |  |  |  |  |
