# Supplementary material for: The baseline distribution of malaria in the initial phase of elimination in Sabang Municipality, Aceh Province, Indonesia
Source: Malar J. 2012 Aug 21;11:291. doi: 10.1186/1475-2875-11-291 (PMC3478225; doi:10.1186/1475-2875-11-291)
Supplement: Additional file 2 — Distribution of respondents by age group. [file 1475-2875-11-291-S2.doc]

Additional file 2. Distribution of respondents by age group

| **Age group (years old)** | **n** | **%** |
| --- | --- | --- |
|  |  |  |
| No Data | 1011 | 6,9 |
| < 5 | 1447 | 9,9 |
| 5 - 10 | 2071 | 14,2 |
| 11 - 20 | 2203 | 15,1 |
| 21 - 30 | 2352 | 16,1 |
| 31 - 40 | 2182 | 15,0 |
| 41 - 50 | 1501 | 10,3 |
| > 50 | 1804 | 12,4 |
| **Total** | **14571** | **100,0** |
|  |  |  |
