# Supplementary material for: The baseline distribution of malaria in the initial phase of elimination in Sabang Municipality, Aceh Province, Indonesia
Source: Malar J. 2012 Aug 21;11:291. doi: 10.1186/1475-2875-11-291 (PMC3478225; doi:10.1186/1475-2875-11-291)
Supplement: Additional file 3 — Descriptive Statistic for Hb level by village. [file 1475-2875-11-291-S3.doc]

Additional file 3. Descriptive Statistic for Hb Level By Village

| **Village** | **Descriptives Statistics for Hb Level (gr/dl)** | | | | | |
| --- | --- | --- | --- | --- | --- | --- |
|  | **Mean** | **95% CI for Mean** | | | **Std. Deviation** |  |
|  |  |  |  |  |  |  |
| Aneuk Laot | 12,78 | 12,64 | - | 12,92 | 1,85 |  |
| Anoi Itam | 12,73 | 12,53 | - | 12,93 | 1,76 |  |
| Balohan | 12,63 | 12,54 | - | 12,73 | 1,71 |  |
| Batee Shok | 12,49 | 12,35 | - | 12,62 | 1,94 |  |
| Cot Abeuk | 12,60 | 12,43 | - | 12,78 | 1,61 |  |
| Cot Ba'u | 12,82 | 12,75 | - | 12,89 | 1,75 |  |
| Iboih | 13,03 | 12,86 | - | 13,20 | 2,02 |  |
| Ie Meulee | 12,70 | 12,60 | - | 12,79 | 1,71 |  |
| Jaboi | 12,53 | 12,35 | - | 12,72 | 1,78 |  |
| Keuneukai | 12,53 | 12,39 | - | 12,68 | 1,60 |  |
| Kota Bawah Timur | 12,74 | 12,64 | - | 12,85 | 1,82 |  |
| Krueng Raya | 12,51 | 12,36 | - | 12,66 | 1,79 |  |
| Paya Keuneukai | 12,74 | 12,52 | - | 12,95 | 1,86 |  |
| Paya Seunara | 12,37 | 12,26 | - | 12,49 | 1,89 |  |
|  |  |  |  |  |  |  |
