# Supplementary material for: The baseline distribution of malaria in the initial phase of elimination in Sabang Municipality, Aceh Province, Indonesia
Source: Malar J. 2012 Aug 21;11:291. doi: 10.1186/1475-2875-11-291 (PMC3478225; doi:10.1186/1475-2875-11-291)
Supplement: Additional file 4 — Distribution of anaemic status by village. [file 1475-2875-11-291-S4.doc]

Additional file 4. Distribution of anaemic status by village

| **Village** | **Haemoglobin Status** | | | | **Total** | |
| --- | --- | --- | --- | --- | --- | --- |
|  | **Anaemic** | | **Non-anaemic** | |  |  |
|  | **n** | **%** | **n** | **%** | **n** | **%** |
|  |  |  |  |  |  |  |
| Aneuk Laot | 278 | 43,2 | 366 | 56,8 | 644 | 100 |
| Anoi Itam | 110 | 40,1 | 164 | 59,9 | 274 | 100 |
| Balohan | 555 | 46,3 | 643 | 53,7 | 1198 | 100 |
| Batee Shok | 347 | 49,3 | 357 | 50,7 | 704 | 100 |
| Cot Abeuk | 163 | 49,8 | 164 | 50,2 | 327 | 100 |
| Cot Ba'u | 1010 | 40,8 | 1466 | 59,2 | 2476 | 100 |
| Iboih | 188 | 36 | 334 | 64 | 522 | 100 |
| Ie Meulee | 570 | 45,6 | 681 | 54,4 | 1251 | 100 |
| Jaboi | 164 | 48,2 | 176 | 51,8 | 340 | 100 |
| Keuneukai | 167 | 46,8 | 190 | 53,2 | 357 | 100 |
| Kota Bawah Timur | 507 | 43,3 | 665 | 56,7 | 1172 | 100 |
| Krueng Raya | 240 | 46,1 | 281 | 53,9 | 521 | 100 |
| Paya Keuneukai | 117 | 47 | 132 | 53 | 249 | 100 |
| Paya Seunara | 474 | 50,6 | 462 | 49,4 | 936 | 100 |
| **Total** | **4890** | **44,6** | **6081** | **55,4** | **10971** | **100** |
|  |  |  |  |  |  |  |
