# Supplementary figures and images for: The baseline distribution of malaria in the initial phase of elimination in Sabang Municipality, Aceh Province, Indonesia
Source: Malar J. 2012 Aug 21;11:291. doi: 10.1186/1475-2875-11-291 (PMC3478225; doi:10.1186/1475-2875-11-291)

Additional file 5. Age distribution of participants in MBS divided per villages


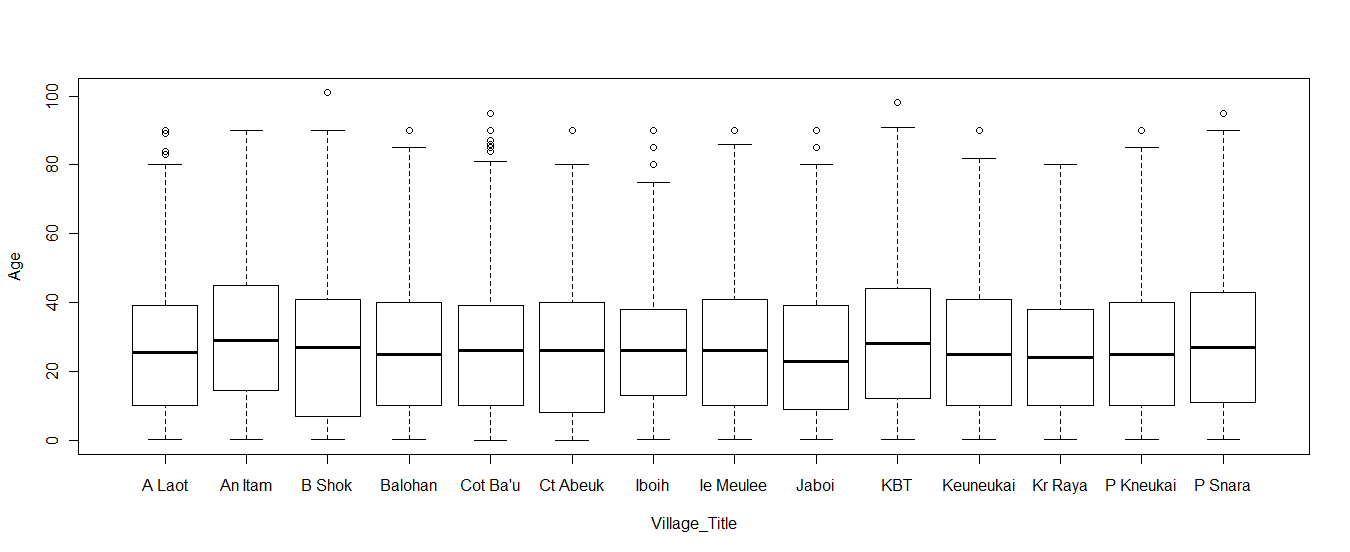

Supplement: Additional file 5 — Age distribution of participants in MBS divided per villages. [file 1475-2875-11-291-S5.doc]
